# Achieving Optimal Esthetics with Immediate Implants and Veneers in the Smile Zone: A Case Study

**DOI:** 10.3390/biomimetics10020105

**Published:** 2025-02-12

**Authors:** Carlos A. Jurado, Jose Villalobos-Tinoco, Daniel Alejandro Montealvan-Aguilar, Silvia Rojas-Rueda, Kiarash Karimi, Nicholas G. Fischer

**Affiliations:** 1 Division of Operative Dentistry, Department of General Dentistry, Health Science Center, College of Dentistry, The University of Tennessee, Memphis, TN 38163, USA; 2 School of Dental Medicine, Ponce Health Sciences University, Ponce 00716, Puerto Rico; 3 Department of Restorative Dentistry, Centro de Estudios Odontologicos (CEO), Queretaro 76050, Mexico; josvt_7@hotmail.com; 4 Independent Researcher, Culiacan 80030, Mexico; 5 School of Dentistry, Autonomous University of Queretaro, Queretaro 76010, Mexico; 6 Division of Dental Biomaterials, Department of Clinical and Community Sciences, School of Dentistry, The University of Alabama at Birmingham, Birmingham, AL 35233, USA; srojasru@uba.edu; 7 Section of Restorative Dentistry, School of Dentistry, University of California Los Angeles, 714 Riverton Av., Los Angeles, CA 90024, USA; kiarash1985@hotmail.com; 8 MDRCBB, Minnesota Dental Research Center for Biomaterials and Biomechanics, University of Minnesota, Minneapolis, MN 55455, USA

**Keywords:** immediate implants, veneers, esthetic dentistry

## Abstract

Background: This case report outlines the clinical workflows for immediate implant placement for both maxillary central incisors and ceramic laminate veneers for the remaining teeth in the smile zone. Methods: The patient’s chief complaint was to improve her smile and address periapical infections with purulent exudate at the apex of her central incisors. Clinical and CBCT evaluations determined that the maxillary central incisors were non-restorable, while the lateral incisors and canines showed signs of incisal wear. Atraumatic extractions were performed for the central incisors, and immediate implants were placed with a 3D-printed surgical guide in conjunction with an autogenous soft tissue grafting procedure. Once the soft tissue between the central incisors was contoured with provisional implant restorations, minimally invasive veneer preparations were performed for porcelain laminate veneers. Final restorations were bonded under dental dam isolation. Results: Single immediate implants for maxillary central incisors can be successfully paired with ceramic laminate veneers on adjacent teeth in the smile zone to replace non-restorable teeth in the esthetic zone. Conclusions: Atraumatic tooth extraction, 3D implant planning with grafting procedures, and minimally invasive ceramic veneers can help in meeting patients’ esthetic and functional expectations. Total isolation using a dental dam maximizes the bonding performance of ceramic restorations.

## 1. Introduction

Dental care in the smile zone is often more complex and challenging than in other areas of the mouth because minor imperfections are easily noticed by patients [1]. Clinicians must carefully assess factors such as the smile line, the number of visible teeth, whether gingival tissues are displayed, tooth position, and tooth shape [2]. Patient expectations must be clearly established, and the clinician should communicate any limitations before performing irreversible procedures to avoid potential complaints. The case complexity is heightened if tooth extractions are necessary, as the periodontium biotype and bone structure must be thoroughly assessed before performing precise, atraumatic extractions [3].

Immediate implant therapy in the esthetic zone is challenging because if the implant is placed incorrectly, or with inadequate surgical technique, it can compromise the surrounding bone, leading to bone loss. In addition, improper contours of the implant prosthesis can hinder proper oral hygiene, which may result in plaque accumulation, bone loss, and ultimately peri-implantitis. Immediate implant therapy involves replacing non-restorable teeth with implants placed directly into fresh extraction sockets immediately following tooth extractions [4]. This approach offers several advantages, including reducing the number of surgical interventions, shortening the overall treatment time, avoiding the use of removable interim prostheses, and delivering predictable results [5,6]. However, achieving favorable esthetic outcomes with implant therapy in the maxillary anterior region requires meticulous management of both the soft and hard tissues. Three-dimensional (3D) planning with CBCT scans provides invaluable information, such as the width of the buccal plate and any need for bone grafting, allowing the clinician to anticipate implant dimensions, including diameter and width [7]. Additionally, advancements in 3D printing technology enable the fabrication of precise surgical guides prior to tooth extraction, improving procedural accuracy [8].

Ceramic laminate veneers in the smile zone are a conservative treatment option that can meet both functional and esthetic demands [9,10]. Veneers are less invasive, requiring the removal of only 3 to 30% of tooth structure, compared to 63 to 72% for traditional full-coverage crowns [11]. Furthermore, veneer restorations have demonstrated excellent long-term success, with feldspathic porcelain reporting a 95% survival rate after 10 years of clinical use [12], and lithium disilicate veneers showing a 97.4% survival rate over the same period [13].

Combining immediate implant therapy and ceramic laminate veneers in the esthetic zone is a challenging clinical scenario. Unfortunately, there is a lack of clinical reports involving the simultaneous use of two immediate implants to replace maxillary central incisors, along with grafting procedures and ceramic veneers for all teeth visible in a wide smile. Therefore, the objective of this report is to outline a comprehensive workflow that includes atraumatic tooth extraction of both central maxillary incisors, immediate implant placement using a 3D-printed surgical guide, the establishment of ideal soft tissue contours though implant provisional restorations, and the placement of ceramic laminate veneers from the maxillary right first molar to the left first molar, using a bonded protocol under rubber dam isolation.

## 2. Case Report

**Figure 1:**
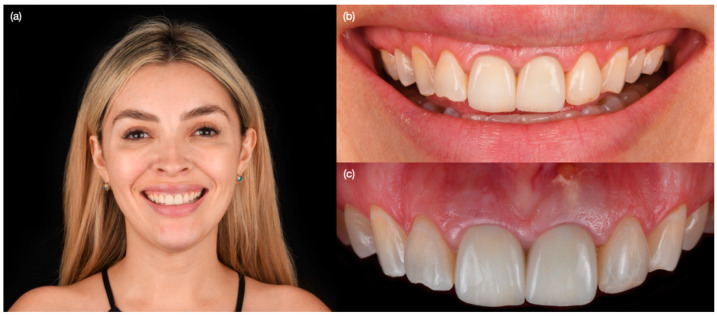
Initial situation. (**a**) Face smiling, (**b**) smile, and (**c**) intra-oral.

**Figure 2:**
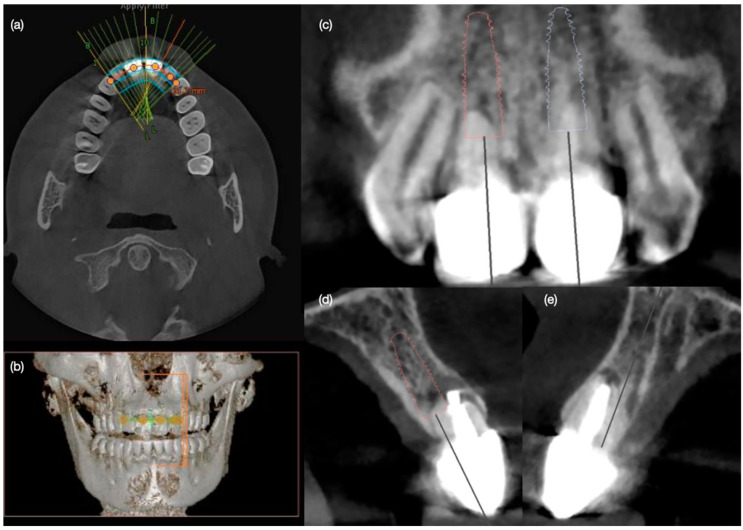
Radiographic evaluation of the central incisors with infection at the apical area. (**a**) CBCT occlusal view, and (**b**) frontal view; (**c**) frontal view and (**d**) and (**e**) lateral views of central incisors.

A 30-year-old female patient presented to the clinic with the chief complaint of having some exudated tissue appearing in the upper anterior area of her mouth, and also disliking her smile (Figure 1). Patient stated that she had previous dental care in another city, including endodontic treatment and crowns for both maxillary central incisors 5 years prior. The patient had undergone extraction of both maxillary first premolars followed by orthodontic treatment 10 years ago. After clinical, photographic, radiographic, and cone beam computed tomography (CBCT) evaluation, the maxillary incisors were diagnosed with chronic periapical abscesses. Other findings included incisal wear for the right canine, right lateral incisor, and left canine, and a stained old composite restoration on the incisal edge of the left lateral incisor. The patient noted that the wear was a significant esthetic concern. Moreover, both central incisors presented grade II mobility. Due to the chronic periapical abscess in both central incisors, the long metal posts, mobility, and apical resorption, the teeth were determined to be non-restorable, and extractions were recommended (Figure 2). The patient was offered different treatment options for the replacement of both central incisors, such as a fixed dental prosthesis from lateral incisor to lateral incisor, a removable partial dental prosthesis, and implant therapy. The patient was also offered minimally invasive porcelain veneers for all her teeth displayed while smiling, from the left first molar to the right first molar, based on her esthetic concerns of wear. Resin composite veneers were similarly offered, and compared and contrasted with porcelain veneers. The patient preferred to have implants, and accepted to have porcelain veneers for the remaining anterior teeth, in order to improve the entire smile. The patient was informed about the option of immediate implant therapy or two-staged implant surgery, and she opted for immediate implant therapy in order to decrease the number of surgical procedures.

**Figure 3:**
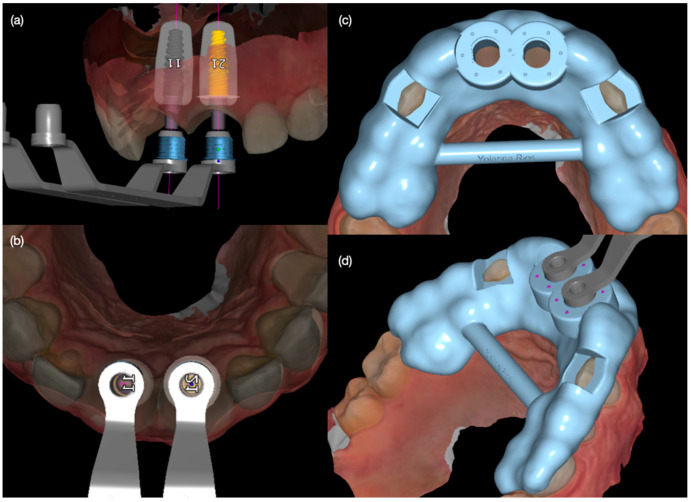
Digital implant planning. (**a**) Frontal view and (**b**) incisal view for both implants, and (**c**) incisal and (**d**) lateral view of surgical guide.

**Figure 4:**
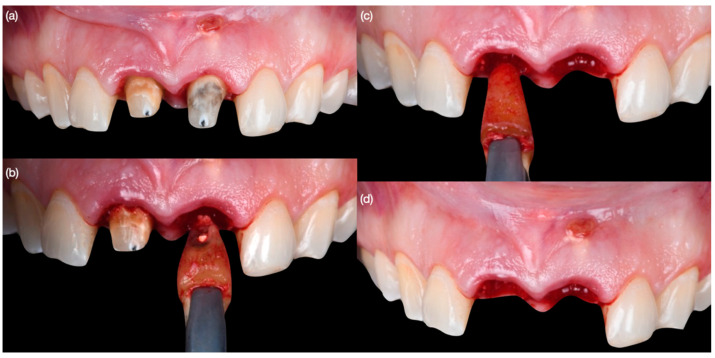
Atraumatic tooth extractions. (**a**) Crowns removed, (**b**) extraction of left central incisor, (**c**) extraction of right central incisor, and (**d**) both teeth extracted.

**Figure 5:**
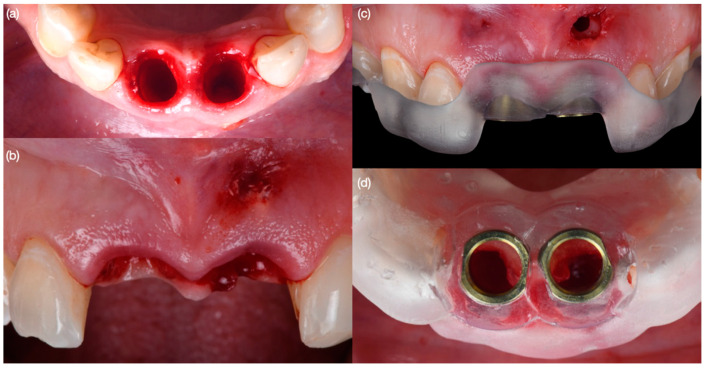
Cleaning of abscess and implant placement. (**a**) Initial situation after extraction—occlusal view and (**b**) frontal view; (**c**) cleaning of abscess and guide placement—frontal view; and (**d**) surgical guide in position—occlusal view.

**Figure 6:**
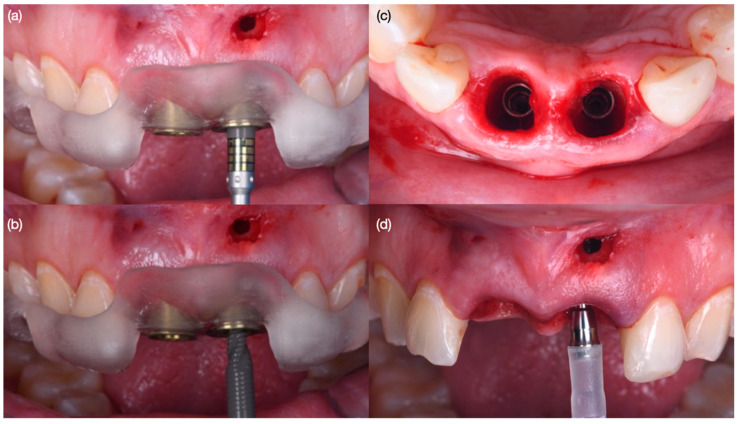
Implant placement. (**a**) initial drilling for implant on left central incisor site, (**b**) placement of implant on left central incisor site, (**c**) both implants placed—occlusal view and (**d**) frontal view.

**Figure 7:**
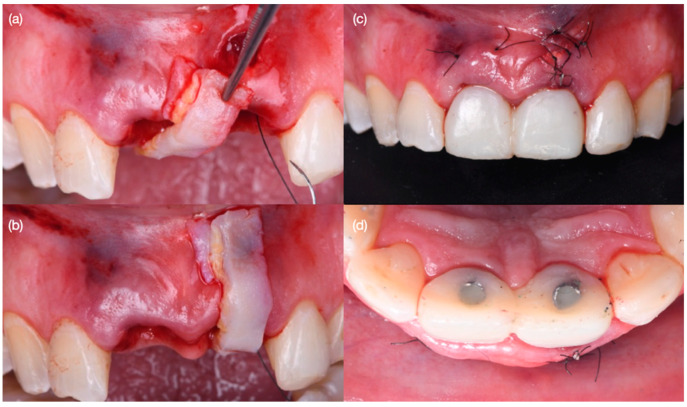
Grafting procedure and provisionals. (**a**) Positioning of autogenous soft tissue graft, (**b**) evaluation of size of position of soft tissue graft, (**c**) screw-retained provisional restorations and grafting procedure—frontal view and (**d**) incisal view.

**Figure 8:**
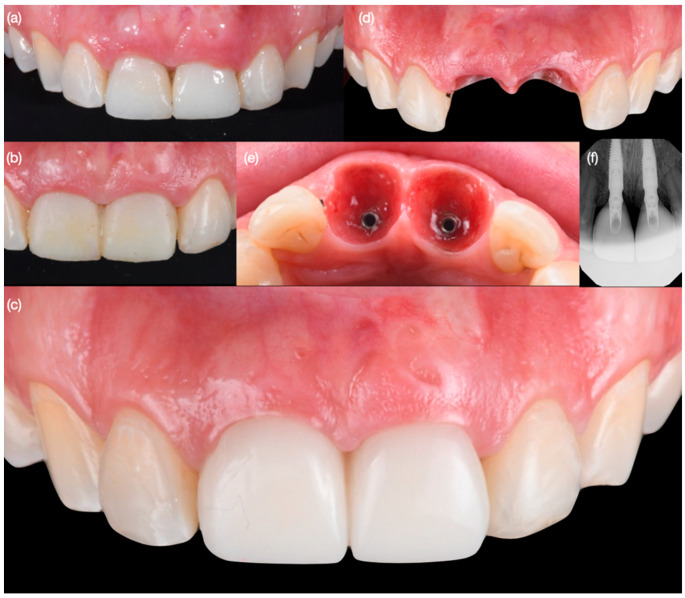
Soft tissue contouring at (**a**) 1 month after surgery, (**b**) recontoured provisional restoration at 2 months, (**c**) new provisional restoration at 3 months, (**d**) soft tissue after 3 months—frontal view and (**e**) incisal view, and (**f**) implant radiographs at 3 months.

**Figure 9:**
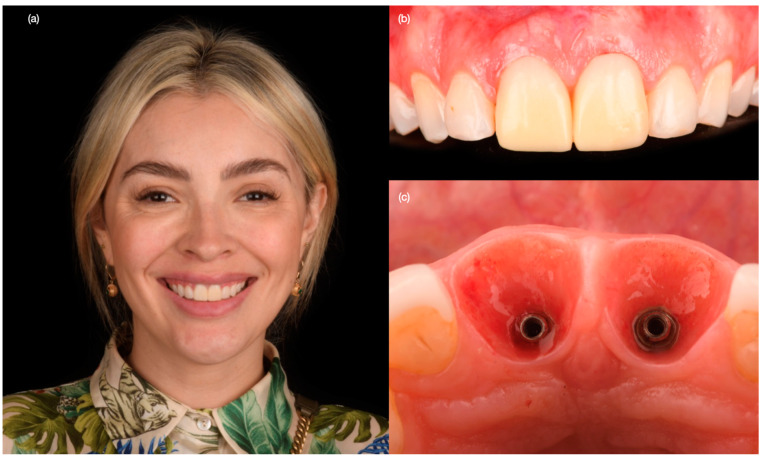
Final implant provisional restoration. (**a**) Face smiling, (**b**) frontal view of provisional restorations, and (**c**) soft tissue contoured.

Diagnostic three-dimensional digital scans (iTero Lumina, San Jose, CA, USA) of the maxilla and mandible were taken, and a traditional facebow record (Artex Facebow, Amann Girrbach, Koblach, Austria) was obtained. Printed models (Phrozen Sonic Mini 8K Resin 3D Printer, Phrozen Technology, Hsinchu City, Taiwan) were then fabricated and mounted on a semi-adjustable articulator (Artex CR, Amann Girrbach, Koblach, Austria). A diagnostic wax-up was performed and mounted in the articulator. The patient also received an intra-oral mock-up with a putty index guide (Elite P&P, Zhermack, Badia Polesine, Italy). An initial CBCT scan and the intra-oral scan were used to digitally plan the implant placement (Exocad DentalCAD, Darmstadt, Germany) and print the surgical guide (Phrozen Sonic Mini 8K Resin 3D Printer, Phrozen Technology, Hsinchu City, Taiwan) (Figure 3). Atraumatic tooth extractions were performed for both maxillary central incisors with the use of a periotome (Anterior Straight PT6, Hu-Friedy, Chicago, IL, USA) and forceps (Upper Anterior Atlas FAF1IS, Hu-Friedy, Chicago, IL, USA), only providing vertical forces, and avoiding any horizontal force (Figure 4). The extraction sockets were cleaned thoroughly with curettes (Universal Curette, Hu-Friedy, Chicago, IL, USA), and the abscess cavity was irrigated with saline solution and chlorohexidine to flush out all the debris and blood. The 3D-printed surgical guide was placed in position, osteotomies were performed, and two implants (3.5 mm diameter; 11.50 mm length, Helix GM, Neodent, Basel, Switzerland) were placed at the planned depth (Figure 5 and Figure 6). An autogenous soft tissue graft was harvested from the palate and positioned on the facial surface of the maxillary left side implant using polyethylene sutures. Interim abutments (Gm Temporary Abutments for Crown, Neodent, Basel, Switzerland) were placed with screw-retained non-occluding interim crowns (Figure 7). The patient had regular follow-up evaluations at 2, 4, 7, and 10 weeks, and at 3 and 4 months, after the implant placement. The provisional restoration was recontoured at week 10, and new sets of interim restorations were made at 3 and 4 months (Figure 8). The patient and clinicians were fully satisfied with the contour obtained with the last set of provisional restorations at 4 months, and it was decided to continue the final stage of the treatment (Figure 9).

**Figure 10:**
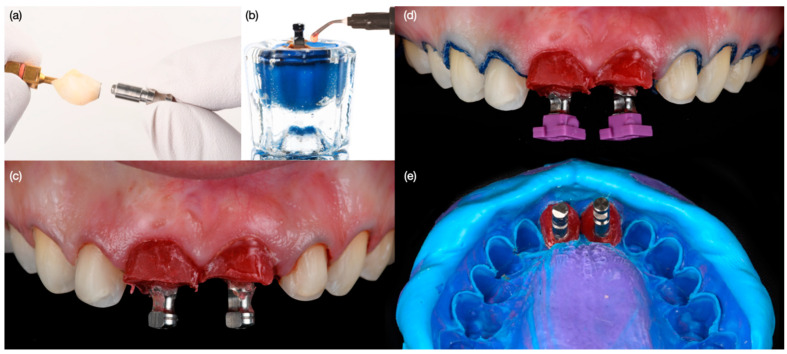
Final impression for implants and veneers. (**a**) Placing impression post for provisional restoration, (**b**) contouring impression post following contours of provisional restorations, (**c**) placement of customized impression posts, (**d**) cord packed for veneer impression and contoured impression posts, and (**e**) final impression performed.

**Figure 11:**
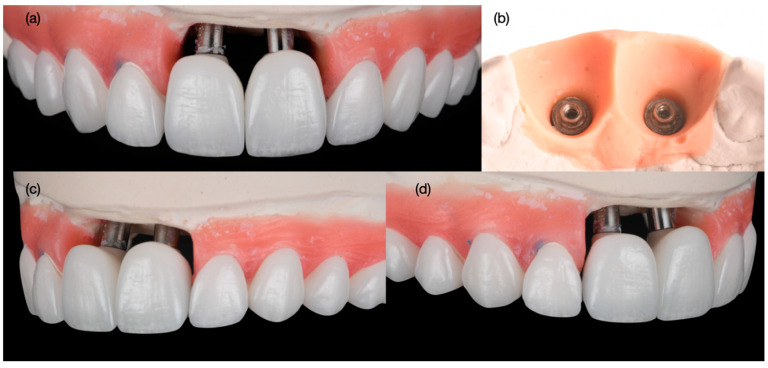
Final restorations fabrication. (**a**) Frontal view of restorations, (**b**) soft tissue contour in master cast, and (**c**) left and (**d**) right side view of restorations.

Minimally invasive veneer preparations were performed with a specialized bur kit (Solution Laminate Veneer Preparation System, Brasseler, Savannah, GA, USA). The provisional restorations were removed, analogs were attached to them, and then they were submerged into a dappen dish full of polyvinyl siloxane material, in order to capture the contours. The impression post was inserted and self-curing acrylic material (Pattern Resin, GC, Tokyo, Japan) was placed around it to obtain a replica of the sub- and supra-gingival contours, and finally the customized impression posts were attached to the implants. A retraction cord (#00, Ultrapak, Ultradent, South Jordan, UT, USA) was placed around the prepared teeth; then, the final preparations were polished with coarse, medium, and fine polishing discs (Sof-Lex XT Disc, 3M, St Paul, MN, USA). Final tooth preparations and implant impressions were made with polyvinyl siloxane material (Elite P&P, Zhermack, Badia Polesine, Italy) (Figure 10). Master casts were created with type IV stone (Fujirock, GC, Tokyo, Japan), and were scanned in order to create the customized implant abutments from zirconia with a titanium base; the design of the abutment was tooth-shaped, with space for a facial veneer. All laminate veneers were hand-crafted from feldspathic porcelain (Noritake Super Porcelain, Kuraray Dental, Tokyo, Japan) (Figure 11).

**Figure 12:**
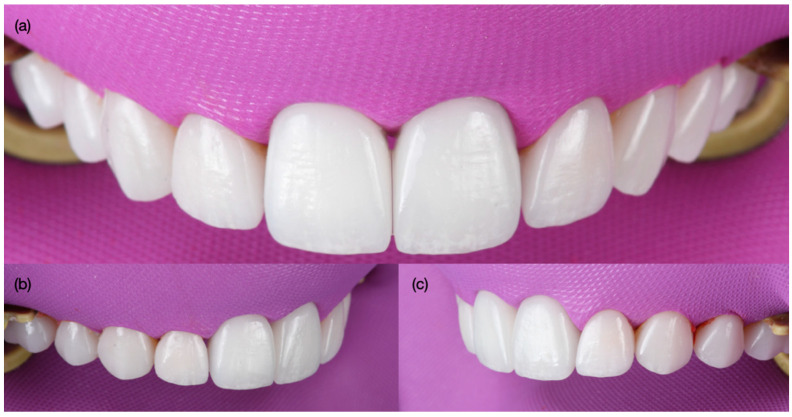
Bonding final restorations under dental dam isolation. (**a**) Frontal, (**b**) right side, and (**c**) left side view.

**Figure 13:**
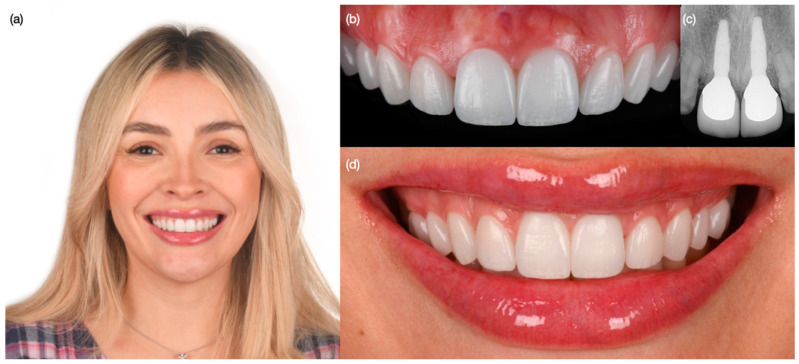
Final bonded restorations. (**a**) Face smiling, (**b**) frontal view of restoration, (**c**) radiograph, and (**d**) smile.

**Figure 14:**
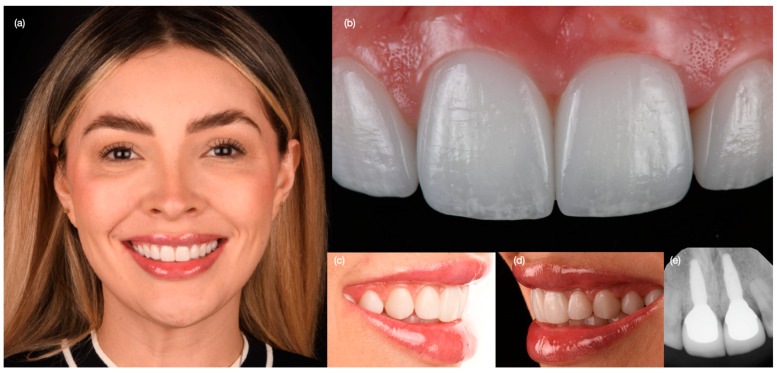
Three-year follow-up. (**a**) Face smiling, (**b**) close-up of central incisor implants, (**c**) right and (**d**) left side view of smile, and (**e**) radiographs of osseointegrated central incisor implants.

A dry try-in of the laminate veneers was performed in order to evaluate the fit, and an additional try-in with paste (Choice 2 Try-In Paste, Milky Bright, Bisco Dental, Schaumburg, IL, USA) was also performed in order to evaluate the final shade. The patient approved the try-in and requested to continue with the cementation. Complete isolation was provided with a dental dam (Flexi Dam, Coltene Holding, Altstatten, Switzerland) from the maxillary right second molar to the left second molar. The dental dam was retained with clamps (Clamp #00, Hu-Friedy, Chicago, IL, USA), in order to achieve proper isolation. The intaglio surface of all the ceramic veneers was treated first with 9.5% hydrofluoric acid (Porcelain Etch, Bisco Dental, Schaumburg, IL, USA) for 60 s, followed by rinsing and air drying, then silane (Bis-Silane, Bisco Dental, Schaumburg, IL, USA) was applied for 60 s, and finally adhesive (All Bond Universal, Bisco Dental, Schaumburg, IL, USA) was applied and the excess was removed with air. The teeth first had sandblasting treatment with water and 29 µm aluminum oxide particles (AquaCare, Velopex International, London, UK); then, the prepared teeth were treated with 32% phosphoric acid (Uni-Etch, Bisco Dental, Schaumburg, IL, USA) for 15 s, then gently air dried, followed by adhesive (All Bond Universal, Bisco Dental, Schaumburg, IL, USA) application, and the excess was removed. Finally, resin cement (Choice 2 Veneer Cement, Milky Bright, Bisco Dental, Schaumburg, IL, USA) was applied, the veneer restorations were bonded to the teeth and implant abutments, and the excess was removed. Each of the ceramic restorations was light-cured on the facial, incisal, mesial, and distal for 20 s each. Excess cement was removed (Figure 12). Dental dam was removed and occlusion was evaluated and adjusted as needed. The patient was pleased with the shape, contours, and shade of the ceramic restorations (Figure 13). An occlusal guard was provided in order to protect the restorations at night. At the 3-year follow-up, the patient was still pleased with the clinical and functional outcome (Figure 14), and no signs of peri-implant diseases were noted.

## 3. Discussion

Dental care in the smile zone presents a challenging clinical scenario for clinicians. Proper planning, including a thorough review of the patient’s medical and dental history, initial diagnostic records such as photographs and dental models, and attentive listening to the patient’s chief complaint, is essential for accurate diagnosis and to outline the possibilities and limitations of the proposed treatment. Dental photography is a valuable tool for documenting the initial condition and monitoring progress throughout treatment [14]. It also allows patients to identify desired changes in their smile, and serves as an ideal communication medium with dental technicians to convey the current tooth shade and desired restoration outcomes [15]. The present report utilized dental photography to document the initial condition, analyze the smile zone, communicate the restoration shade to the dental technician, and compare the initial state with the final outcome.

Alveolar bone loss in height and width following tooth extraction is a significant concern for subsequent implant restorations, as it can compromise esthetics [16,17]. It is widely recognized that the technique used for tooth extraction affects the degree of alveolar bone loss. Traditional extraction methods involve elevators, luxators, and forces that often result in expansion of the socket dimensions, and can cause fractures or deformities in the interproximal bone, making it difficult to preserve the integrity of the socket. These conventional methods also damage the surrounding soft tissues, including the interdental papillae, which complicates successful implant placement and create challenges for future prosthetic restoration [18,19]. Atraumatic tooth extractions, which avoid flap elevation and employ vertical force to lift the tooth, minimize damage to surrounding soft and hard tissues [20]. Studies indicate that this technique improves ridge preservation compared to traditional methods. Factors such as patient behavior, tooth morphology, and surgical technique influence soft and hard tissue loss, but the extraction technique is key to better outcomes [18,21,22]. A recent clinical study comparing flap and flapless extraction techniques found that flapless methods yielded superior bone preservation, with significantly less vertical bone loss, and authors concluded that the flapless technique offered better results [23]. In the present clinical report, atraumatic flapless tooth extractions of both maxillary central incisors were performed to minimize tissue damage before immediate implant therapy.

Immediate implant therapy involves placing an implant into a freshly extracted socket during the same procedure. Previously, clinicians waited 12 months or longer for socket healing before implant placement. Immediate implants expedite treatment, reduce the number of surgeries, and aim to maintain the original gingival architecture [24]. Moreover, this timing for implant placement has shown high survival rates. A systematic review on the survival and success rate of implants placed immediately into fresh extraction sockets was performed. The authors assessed 46 prospective studies, with a mean follow-up of 2 years; the factors analyzed were the reasons for tooth extraction, antibiotic use, the position of the implant, and the type of loading. The results displayed a 98.4% survival rate, and the authors concluded that high survival rates were observed for immediate implant therapy [25]. Similarly, a recent retrospective case series study evaluated the treatment outcome of 36 immediate implants placed in fresh extraction sockets, with follow-up visits at 1, 5, 15, 20, and 22 years after prosthetic loading, and the results displayed a survival rate of 97.2% during the 22-year follow-up. Moreover, the marginal bone loss from baseline to 22-year follow-up was 1.61 mm, and the authors concluded that immediate implant placement offers excellent prognosis [26]. These results highlight the predictability and success of immediate implant therapy like that implemented in this report.

The immediate implants placed in this case study were positioned with the aid of a surgical guide to achieve greater accuracy compared to traditional freehand placement. A recent in vitro study compared the accuracy of freehand versus surgical guide implant placement among experienced and non-experienced dental implant practitioners. In the study, ten clinicians were divided into experienced and non-experienced groups, and placed a total of 60 implants in 20 acrylic resin models. The results showed that the accuracy of implants placed with a surgical guide was significantly higher than that of freehand placement. The authors concluded that a surgical guide is a valuable tool for precise implant positioning [27]. Additionally, a systematic review and meta-analysis evaluated the accuracy of fully guided, half-guided, and freehand implant placement. The review analyzed 10 randomized clinical trials, assessing coronal and apical deviation in implant positioning. The findings concluded that fully guided placement offered the highest accuracy, followed by half-guided placement, while freehand placement showed the least accuracy [28]. That said, drawbacks of immediate implants include an increased risk for surgical complications compared to conventional protocols, more rigid case selection criteria for successful outcomes, more complex peri-implant soft tissue management, and a higher need for patient care compliance [29]. 

Immediate implant therapy was performed in conjunction with soft tissue grafting. While this approach may involve more clinical work, the literature shows that it has positive outcomes when performed correctly. A recent systematic review and meta-analysis examined the use of connective tissue grafts (CTGs) in immediate implant placements. The study reviewed publications on patients who required simultaneous or delayed CTGs, and evaluated both the benefits and drawbacks of the procedure. It concluded that CTGs associated with immediate implants can help to maintain gum levels, but do not increase volume. CTGs are thus considered favorable for achieving successful esthetic outcomes in immediate implant placement [30]. Another recent systematic review and meta-analysis investigated the impact of soft tissue augmentation on clinical and radiographic outcomes following immediate implant placement and provisionalization. This review compared publications on immediate implants placed with and without CTGs. The results indicated that facial soft tissue thickness increased significantly in patients who underwent the graft procedure. In contrast, the control group, which did not receive grafting, experienced significantly higher marginal bone loss. The authors concluded that using a graft significantly reduces marginal bone loss and vestibular recession, while increasing soft tissue thickness [31]. Given the documented benefits of grafting procedures in immediate implant placement, simultaneous soft tissue grafting was incorporated into the treatment plan for this case report.

The literature extensively documents the use of feldespathic porcelain veneers to fulfill patients’ esthetic and functional demands [32,33,34]. Moreover, positive optical properties and long-term survival rates have been reported for laminate veneers fabricated from feldspathic porcelain. A study evaluated the clinical performance of 318 porcelain veneers placed in 84 patients for 5, 10, and 20 years in service. The restorations were assessed for their esthetics, marginal discoloration, and integrity. The results showed survival rates of 94.4% after 5 years, 93.5% at 10 years, and 82.9% at 20 years, and the authors concluded that porcelain veneers offer predictable and successful restoration with high survival rates [35]. Advances in technology now allow dental technicians to hand-craft thinner veneers, enabling clinicians to perform minimally invasive preparations [36,37]. Based on these positive findings, feldspathic porcelain was the material selected for the restorations in this case, due to its excellent esthetic properties and longevity.

Minimally invasive tooth preparations were performed to preserve enamel structure for bonding the ceramic laminate veneers. Studies have shown that resin cement adheres more strongly to enamel than to dentin [38,39]. Bonding the final restorations under total isolation with a dental dam is strongly recommended. Complete isolation offers several advantages, including preventing patient inhalation of toxic materials, avoiding contamination, and ensuring optimal bonding between ceramic restorations and tooth structure [40,41]. Although dental dam use is sometimes criticized for the time required for its placement, studies have shown that it typically requires less than 2 min for its placement [42]. Furthermore, a Cochrane Library review confirmed that dental dams lead to a lower failure rate [43]. Lastly, clinical contamination during restorative procedures has been shown to negatively impact adhesive interfaces. A recent review of the literature concluded that saliva and blood contamination decrease the bond strength of dental adhesives [44]. For these reasons, dental dam isolation was employed during the cementation of the final restorations in this case.

This case study has several limitations, one of which is the small sample size inherent to a single case report. Future studies should include a larger number of patients treated with the same clinical approach in order to obtain more robust results. Additionally, longer follow-up periods are necessary in order to evaluate the long-term effectiveness of the treatment. Different types of dental ceramics, such as zirconia and novel hybrid ceramics, should also be clinically tested to assess the strength of bonded restorations. Furthermore, future research should investigate the most effective immediate implant protocols for positioning the facial bone crest in patients with defective labial bone plates. Additionally, it would be valuable to explore the relationship between long-term mucosal stability and implant outcomes.

## 4. Conclusions

Replacing both of the maxillary central incisors with immediate implants, alongside laminate veneers for the adjacent teeth, poses a complex challenge for clinicians. A flapless, atraumatic tooth extraction minimizes the risk of gingival recession, while the use of a 3D-planned surgical guide ensures precise implant placement. Provisional implant restorations are key in shaping the desired gingival contours, and ceramic laminate veneers further enhance the esthetic outcome. Conducting bonding procedures under dental dam isolation maximizes the performance of the materials, leading to highly satisfactory results at the 3-year follow-up.

## Data Availability

Data are contained within the article.
